# Super-Resolution Imaging of the A- and B-Type Lamin Networks: A Comparative Study of Different Fluorescence Labeling Procedures

**DOI:** 10.3390/ijms221910194

**Published:** 2021-09-22

**Authors:** Merel Stiekema, Frans C. S. Ramaekers, Dimitrios Kapsokalyvas, Marc A. M. J. van Zandvoort, Rogier J. A. Veltrop, Jos L. V. Broers

**Affiliations:** 1 Department of Genetics and Cell Biology, Maastricht University Medical Centre, 6200 MD Maastricht, The Netherlands; m.stiekema@maastrichtuniversity.nl (M.S.); f.ramaekers@maastrichtuniversity.nl (F.C.S.R.); d.kapsokalyvas@maastrichtuniversity.nl (D.K.); mamj.vanzandvoort@maastrichtuniversity.nl (M.A.M.J.v.Z.); 2 GROW-School for Oncology and Developmental Biology, Maastricht University Medical Centre, 6200 MD Maastricht, The Netherlands; 3 Interdisciplinary Center for Clinical Research, IZKF, RWTH Aachen University, 52074 Aachen, Germany; 4 CARIM-School for Cardiovascular Diseases, Maastricht University Medical Centre, 6200 MD Maastricht, The Netherlands; 5 Institute for Molecular Cardiovascular Research IMCAR, RWTH Aachen University, 52074 Aachen, Germany; 6 Department of Biochemistry, Cardiovascular Research Institute Maastricht, Maastricht University, 6200 MD Maastricht, The Netherlands; r.veltrop@maastrichtuniversity.nl

**Keywords:** nuclear lamins, laminopathy, confocal scanning laser microscopy, STED microscopy, antibodies, transfection, resolution, lamin layer thickness, colocalization

## Abstract

A- and B-type lamins are type V intermediate filament proteins. Mutations in the genes encoding these lamins cause rare diseases, collectively called laminopathies. A fraction of the cells obtained from laminopathy patients show aberrations in the localization of each lamin subtype, which may represent only the minority of the lamina disorganization. To get a better insight into more delicate and more abundant lamina abnormalities, the lamin network can be studied using super-resolution microscopy. We compared confocal scanning laser microscopy and stimulated emission depletion (STED) microscopy in combination with different fluorescence labeling approaches for the study of the lamin network. We demonstrate the suitability of an immunofluorescence staining approach when using STED microscopy, by determining the lamin layer thickness and the degree of lamin A and B1 colocalization as detected in fixed fibroblasts (co-)stained with lamin antibodies or (co-)transfected with EGFP/YFP lamin constructs. This revealed that immunofluorescence staining of cells does not lead to consequent changes in the detected lamin layer thickness, nor does it influence the degree of colocalization of lamin A and B1, when compared to the transfection approach. Studying laminopathy patient dermal fibroblasts (*LMNA* c.1130G>T (p.(Arg377Leu)) variant) confirmed the suitability of immunofluorescence protocols in STED microscopy, which circumvents the need for less convenient transfection steps. Furthermore, we found a significant decrease in lamin A/C and B1 colocalization in these patient fibroblasts, compared to normal human dermal fibroblasts. We conclude that super-resolution light microscopy combined with immunofluorescence protocols provides a potential tool to detect structural lamina differences between normal and laminopathy patient fibroblasts.

## 1. Introduction

The nuclear lamins together with the nuclear lamin-associated proteins form the nuclear lamina, which is part of the nuclear envelope (NE) [1,2,3]. Lamins are type V intermediate filament (IF) proteins and are grouped into A- and B-type lamins, encoded by three different genes. *LMNA*, located on chromosome 1, encodes for the major isoforms lamin A and lamin C, for the germ cell-specific isoform lamin C2, and for the isoform lamin A∆10. *LMNB1*, located on chromosome 5, encodes for lamin B1, while *LMNB2*, located on chromosome 19, encodes for lamin B2 and lamin B3, a germ cell-specific isoform that arises by alternative RNA splicing. Lamins A and C are mainly expressed in differentiated cells, while the B-type lamins show a more ubiquitous tissue and cell distribution [1,4,5].

Both A- and B-type lamins are composed of an N-terminal head domain, a long α-helical coiled-coil central rod domain, and a globular C-terminal tail. The C-terminal domain contains a nuclear localization signal and an immunoglobulin fold, including a CaaX motif (C: cysteine, a: aliphatic residue, X: any residue). The latter is important in their post-translational modifications [5,6]. The lamins form higher-order polymers, the formation of which is initiated by homodimerization of the separate lamin subtype monomers. Subsequently, heterotetramers are formed, which can be composed of both A- and B-type lamins and are found to be about 3.5 nm in diameter [7,8]. This polymer formation is followed by anti-parallel interaction of the polymers, leading to protofilaments. These in turn further assemble into ~14 nm thick IF-like filaments [4,5,9].

A-and B-type lamins are not only localized in the NE, but also in the nucleoplasm, where they assemble into different structures, such as distinct foci, intranuclear and trans-nuclear channels, and nucleoplasmic veil-like structures [10]. The intra- and trans-nuclear channels are thought to be part of the nucleoplasmic reticulum, a larger network of penetrating and branching invaginations of the NE [11,12].

Lamins can bind a wide variety of proteins, called lamin-binding proteins. These include structural proteins, signaling molecules, and transcription factors [5]. The nuclear lamina is also connected to the cytoskeleton via the transmembrane linker of the nucleoskeleton and cytoskeleton (LINC) complexes, which transmit the forces generated by the cytoskeleton to the nucleoplasm [13]. As could be expected from the numerous lamin-binding proteins and the classification of lamins as type V intermediate filament proteins, lamins are involved in a broad range of functions such as providing structural support to the cell [14,15,16,17,18,19], chromatin organization [18,20,21,22,23,24], apoptosis [25], and mitosis [9,26]. Furthermore, accumulating evidence points towards the role of the lamins in mechano-sensing and the mechano-response of cells [27,28,29,30,31].

Mutations in the genes encoding nuclear lamins cause several diseases, for example, Emery–Dreifuss muscular dystrophy (EDMD), type II Dunnigan-type familial partial lipodystrophy, and the Hutchinson–Gilford progeria syndrome [32]. These rare diseases are termed laminopathies and are mostly caused by *LMNA* mutations. Only a few mutations in the genes encoding for B-type lamins or lamin-associated proteins have been reported [32,33,34,35]. The laminopathies can be classified into those that affect striated muscle, those that affect adipose tissue, those involving peripheral nerve systems, or those affecting multiple systems with signs of accelerated aging (with some overlap between affected organs and tissues) [1].

Cells obtained from these patients show aberrations in the localization of each lamin subtype at the nuclear lamina, resulting in nuclear herniations, honeycomb-like structures, and even donut-shaped nuclei [36,37,38]. However, only a minor fraction of diseased cells shows these readily visible abnormalities. Up to 90% of laminopathy cells can have a normal appearance by visual microscopy screening [36,37,38], making it difficult to classify cultures based on nuclear abnormalities only [37]. Conclusively, these visible abnormalities are most probably only the tip of the iceberg of lamina disorganization in laminopathy cells. Moreover, the exact pathophysiology of the lamin mutations and how these can lead to often tissue-specific disorders remains unclear. Furthermore, lamin protein expression is often dysregulated in cancer and can possibly affect cancer progression [39,40,41]. In addition, several viral infections result in a disrupted lamina in the viral entry and/or egress [42].

To better understand the mechanisms of how lamins contribute to disease it is important to study the lamin network in both healthy and diseased cells. This is traditionally performed using (confocal) immunofluorescence microscopy or electron microscopy. Only a few studies so far have studied the lamin network in greater detail with super-resolution microscopy techniques. Super-resolution microscopy is a collective term for a range of imaging techniques that overcome the Abbe diffraction limit of conventional light microscopy. Previous studies with super-resolution microscopy already indicated that A- and B- type lamins form distinct, yet interacting filamentous networks [43,44,45]. Using three-dimensional structured illumination microscopy (3D-SIM) (resolution ~100 nm) and computational image analysis Shimi et al. [45] demonstrated that lamin A, C, B1, and B2 are present as (partially) distinct meshworks within the lamina of mouse embryonic fibroblast (MEF) nuclei with a large degree of overlap between the networks. The distinct networks for each lamin isoform showed similar physical characteristics, with only small differences in the size of areas devoid of lamins. Xie et al. confirmed these results with photoactivated localization microscopy (PALM)/direct stochastic optical reconstruction microscopy (dSTORM) (resolution ~20 nm) and computational reconstruction, indicating that the lamin filaments are organized as overlapping yet independent networks formed by homo-oligomers of lamin A, C, and B1 [43]. Using two-colour STORM imaging Nmezi et al. [44] found that lamin B1 forms an outer rim within the nuclear lamina, preferentially localizing closest to the inner nuclear membrane (INM), while the lamin A/C network localizes closer to the nucleoplasm. This shift was found to be ~15–20 nm. These findings were confirmed with phasor-assisted Metal Induced Energy Transfer-Fluorescence Lifetime Imaging Microscopy (MIET-FLIM), as reported by Figueiras et al. [46].

To study structures at the nanometer scale with super-resolution microscopy, different labeling approaches can be used. In all the studies mentioned above immunofluorescence staining was used, which has several disadvantages. The combination of both primary and secondary antibodies could cause a decrease in resolution, since these complexes are rather large (~30 nm), and linkage errors could occur, due to the distance between the fluorescent dye (on the secondary antibody) and the actual localization of the protein [47,48,49]. It is obvious that both problems are less prominent when using transfection, since Green Fluorescent Protein (GFP) (derived) molecules are only ~3–4 nm in size. However, the major advantage of using antibodies is that staining laminopathy patient fibroblasts with antibodies is much more convenient than using transfection, due to the low transfection efficiency in these patient fibroblasts. Furthermore, while transfection in principle allows imaging of living cells, one should be aware that adding a fluorescent protein could modify the level of expression, activity, and localization of the protein of interest [48,50]. Notably, as described, most super-resolution microscopy studies on the nuclear lamina network make use of the indirect immunofluorescence technique with primary-secondary antibody labeling and still achieve a resolution that allows visualization of differences in the A- and B-type lamin networks when imaging co-labeled cells [43,44,45,46,51,52].

In the underlying study, both Stimulated Emission Depletion (STED) microscopy [53,54] and Confocal Scanning Laser Microscopy (CSLM) are used to study the nuclear lamina of healthy and laminopathy fibroblasts. The advantages of STED over other super-resolution microscopes used for studying the nuclear lamina are that it offers fast acquisition, does not require extensive post-processing of the images, offers 3D sectioning options, and can be combined with relatively easy staining protocols, avoiding the need for specialized buffers which limit in vivo applications [54,55,56]. As a result, STED allows live and fixed cell imaging at any level of the nucleus.

Using confocal and STED microscopy, the lamina layer thickness and the degree of lamin A(/C) and lamin B1 colocalization were determined in mouse 3T3 fibroblasts and normal human dermal fibroblasts (nHDF). For comparison of the effective resolution differences between different labeling methods (immunofluorescence vs. (co-)transfection), we have used mouse 3T3 fibroblasts. Since no obvious differences in resolution between these methods were found in either confocal and STED microscopy, we used immunofluorescence labeling for determining the colocalization between lamin A/C and lamin B1 in a cell culture of laminopathy patient dermal fibroblasts.

Because of the known abnormalities in the lamin A/C network in laminopathy patient dermal fibroblasts, we predicted that even in apparently normal-looking laminopathy cells, the lamin A/C and lamin B1 co-localization would be reduced compared to nHDF cells. As expected these differences were more prominently detected using STED microscopy, suggesting an additional value for STED microscopy to investigate lamina abnormalities in laminopathy patient fibroblasts. Additional studies on a large patient sample cohort should indicate the potential clinical value of this type of analysis.

## 2. Results

### 2.1. Comparison of CSLM and STED Microscopy: Resolution Differences

**Figure 1:**
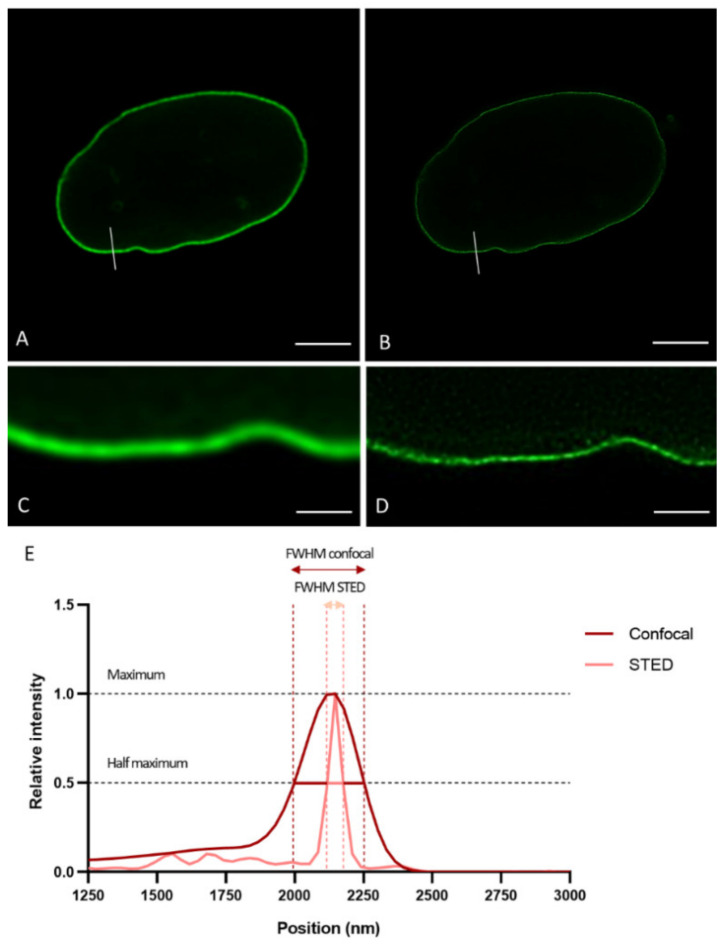
Confocal laser scanning microscopy (**A**) and STED microscopy (**B**) images of nHDF stained with antibodies against lamin B1. The region of interest (ROI) is used for the plot profile displayed in (**E**). Scale bars: 5 µm. (**C**,**D**) Higher magnification (5×) of the region around ROIs in (**A**,**B**). Scale bars: 1 µm. (**E**) Plot profile of lamin intensity, normalized to maximum value. The Full-Width Half Maximum (FWHM) (i.e., the width of the peak at half of its maximum value) of STED (pink) and confocal laser scanning microscopy (red) is indicated.

To demonstrate the resolution differences between CSLM and STED microscopy when studying the lamin network, we plotted intensity profiles of lines drawn perpendicular to the lamina in confocal and STED images acquired in the mid-level of the nucleus (Figure 1A,B). Figure 1 displays a representative example of a confocal and STED image of nHDF stained with antibodies against lamin B1, and the plot profile of a region of interest (ROI) as described above. As expected, the plot profile of the image obtained with confocal microscopy is much broader in comparison to that of the image obtained with STED microscopy (Figure 1E). The intensity visible on the left side of the plot is originating from the lamin signal in the nucleoplasm. The Full-Width Half Maximum (FWHM) (i.e., the width of the peak at half of its maximum value) of these kinds of plot profiles were calculated. In this study, FWHM were determined following the above-described procedure for both 3T3 cells (transfected with fluorescent EGFP- and/or YFP-lamin construct or (co-)stained with antibodies ) and nHDF cells ((co-)stained with antibodies ) (see below) (for every condition and cell type at least n = 7 cells).

The smallest measured FWHM was used as an indication for the resolution. We found a clear difference in effective resolution between confocal and STED in all labeling conditions and for both cell types. The smallest measured FWHM was 241 nm for CSLM and 60 nm for STED microscopy.

### 2.2. Live and Fixed Cells Show Similar Laminopathy Network Structures

**Figure 2:**
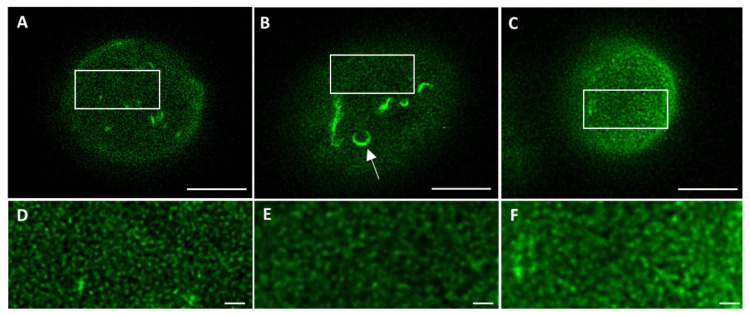
STED microscopy images of the top of the nucleus from (**A**) non-fixed live lam-B1-EGFP transfected 3T3 cells, (**B**) fixed lam-B1-EGFP transfected 3T3 cells, and (**C**) fixed 3T3 cells stained with antibodies against lamin B1. (**D**–**F**) Higher magnification (2.9×) of ROIs (white rectangles) indicated in (**A**–**C**), respectively. Arrow in **B** indicates structures of the nucleoplasmic reticulum. Scale bars: 5 µm.

To exclude the introduction of artifacts upon cell fixation, 3T3 cells transfected with lamin-B1-EGFP were imaged in both live and fixed conditions. Comparing the STED microscopy images of the top of the nucleus demonstrates a similar lamin network in both conditions (Figure 2A,B,D,E), suggesting that no artifacts have been introduced with cell fixation. Next, the same types of images were compared for fixed transfected 3T3 cells and fixed 3T3 cells stained with antibodies (Figure 2B,C,E,F). Apart from the absence of lamin structures belonging to the nuclear reticulum (seen as more bright structures in Figure 2A,B, indicated with an arrow in Figure 2B), a similar lamin network is visualized, suggesting no introduction of artifacts by the transfection procedure.

These observations are based on judgment by eye. To obtain more quantitative information about potential differences in lamin layer thickness and lamin A and B1 colocalization between transfection and antibody staining, determination of the lamina FWHM and colocalization analysis was performed.

### 2.3. Transfection and Immunofluorescence Staining Leads to Comparable Layer Thickness

**Figure 3:**
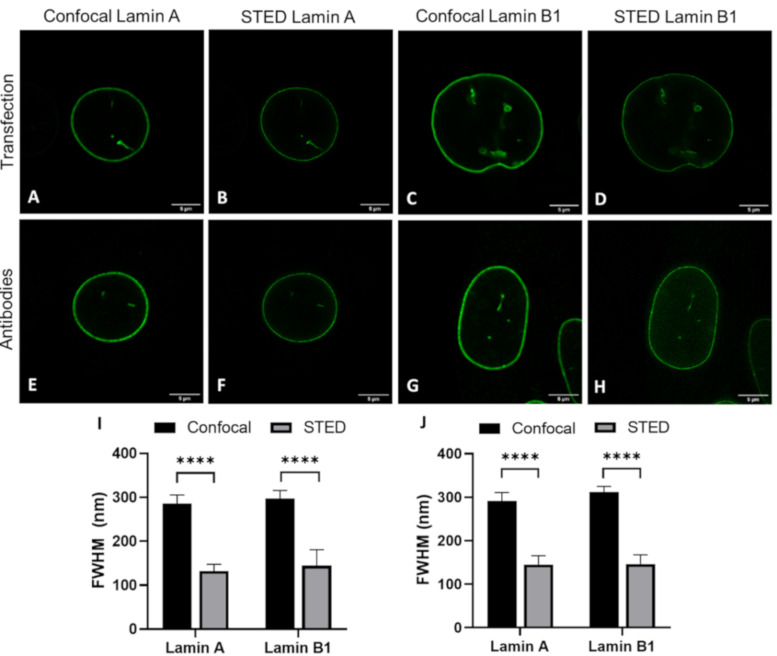
Comparison of confocal (**A**,**C**,**E**,**G**) and STED (**B**,**D**,**F**,**H**) microscopy images. (**A**,**B**) 3T3 lamin-A-YFP transfected. (**C**,**D**) 3T3 lamin-B1-EGFP transfected. (**E**,**F**) 3T3 cells stained with antibodies against lamin A. (**G**,**H**) 3T3 cells stained with antibodies against lamin B1. Scale bars: 5 µm. (**I**,**J**) Average FWHM (nm) of confocal and STED microscopy images of (**I**) transfected 3T3 cells and (**J**) antibody-stained 3T3 cells. Bars, mean ± SD. **** *p* ≤ 0.0001.

The details visible with immunofluorescence staining compared to those seen after transfecting cells with a construct for a fluorescent protein could potentially be different. These differences might influence the observed layer thickness of lamin A and lamin B1, especially when using super-resolution techniques. For this reason, we determined the lamina thickness of 3T3 cells transfected with either lamin-A-YFP or lamin-B1-EGFP (Figure 3A–D,I and Figure S1) and compared those to the values found for 3T3 cells stained with antibodies against either lamin A or B1 (Figure 3E–H,J and Figure S2). For all cells, we additionally compared the lamin layer thickness found in confocal and STED imaging. All results are summarized in Table 1.

**Figure 4:**
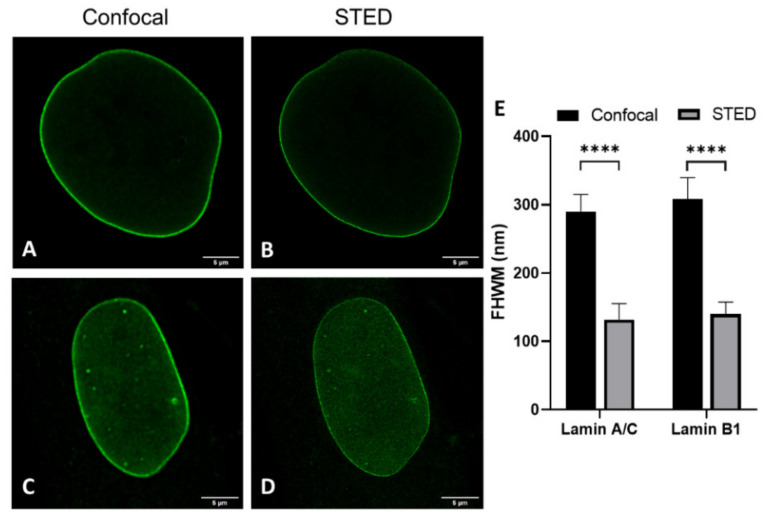
Comparison of confocal (**A**,**C**) and STED (**B**,**D**) microscopy images. (**A**,**B**) nHDF stained with antibodies against lamin A/C. (**C**,**D**) nHDF stained with antibodies against lamin B1. Scale bars: 5 µm. (**E**) Average FWHM (nm) of confocal and STED images of antibody-stained nHDF. Bars, mean ± SD. **** *p* ≤ 0.0001.

Analysis of transfected cells was only performed for 3T3 cells, not for nHDF, due to well-known low transfection efficiencies in the latter cell culture. We compared layer thicknesses of antibody-stained lamin A(/C) (note: in 3T3 cells antibody staining of lamin A in nHDF of lamin A/C) and lamin B1 in nHDFs (Figure 4 and Figure S3) and 3T3 cells (Figure 3E-H,J and Figure S1) to determine the effect of cell type on lamin layer thicknesses (Table 1).

**Figure 5:**
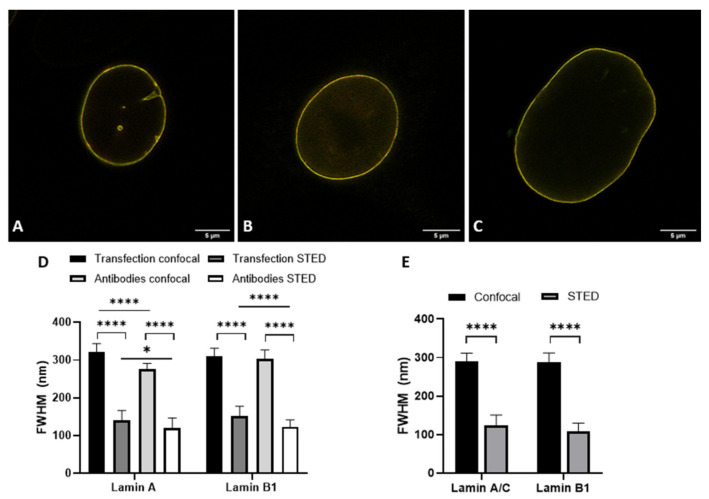
(**A**–**C**) Merged STED microscopy images of lamin A(/C) (red) and lamin B1 (green). (**A**) 3T3 cells transfected with both lamin-A-YFP and lamin-B1-EGFP. (**B**) 3T3 cells stained with antibodies against lamin A and lamin B1. (**C**) nHDF stained with antibodies against lamin A/C and lamin B1. Scale bars: 5 µm. (**D**,**E**) Average FWHM (nm) of confocal and STED microscopy images of co-transfected 3T3 cells and 3T3 cells co-stained with antibodies (**D**), and nHDF stained with antibodies (**E**). Bars, mean ± SD. * *p* ≤ 0.05; **** *p* ≤ 0.0001.

Lastly, the lamin layer thickness of lamin A(/C) and B1 in cells co-labeled with lamin A(/C) and lamin B1 was determined and revealed similar values in the layer thickness of lamin A and B1 with confocal and STED microscopy, independent of either the choice for transfection or antibody staining, or cell type (Figure 5, Table 1).

The most evident observation when comparing the CSLM and STED lamin layer thickness is that the lamin layer thickness for STED microscopy images is significantly decreased as compared to those in confocal microscopy. This is valid under all conditions and in agreement with the difference in resolution between the two techniques.

When comparing the values found for transfection to those for antibody staining a few significant differences can be found. The lamin layer thickness in STED images of 3T3 cells transfected with lamin A is significantly smaller (*p* = 0.004) compared to 3T3 cells stained with antibodies against lamin A. This is also the case for confocal images of lamin B (*p* = 0.001). In contrast, the lamin layer thickness of lamin A in co-stained 3T3 cells is significantly lower in confocal (*p ≤* 0.0001) and STED (*p* = 0.0003) images compared to lamin A in the co-transfected cells. In STED images of these co-stained/co-transfected cells, lamin B1 also demonstrates a significantly lower lamin layer thickness (*p ≤* 0.0001) in the antibody staining condition.

However, we want to stress that the differences, although statistically significant, are minor in view of the standard deviation for the average thickness. Additionally, the differences are not systematically in favor of one parameter. We thus conclude that there is no consistent change in lamin layer thickness in either the transfection or antibody staining condition, indicating that antibody staining does not influence the observed layer thickness of lamin A and B1 when compared to the transfection approach.

### 2.4. Co-Transfection and Immunofluorencent Co-Staining Demonstrate a Similar Degree of Lamin A and B1 Colocalization in 3T3 Cells

To obtain more information about the overlap and correlation between the lamin A and B1 network, the degree of colocalization can be determined by Pearson’s correlation coefficient, which measures the degree of correlative variation of two channels; the higher the value, the more co-dependent both channels are, with a perfect correlation for a value of +1 and perfect anti-correlation for −1 [57]. To determine if different labeling approaches reveal different results for colocalization analysis, the Pearson’s correlation coefficient was determined in both lamin A and lamin B1 co-transfected 3T3 cells and 3T3 cells co-stained with antibodies against lamin A and B1.

**Figure 6:**
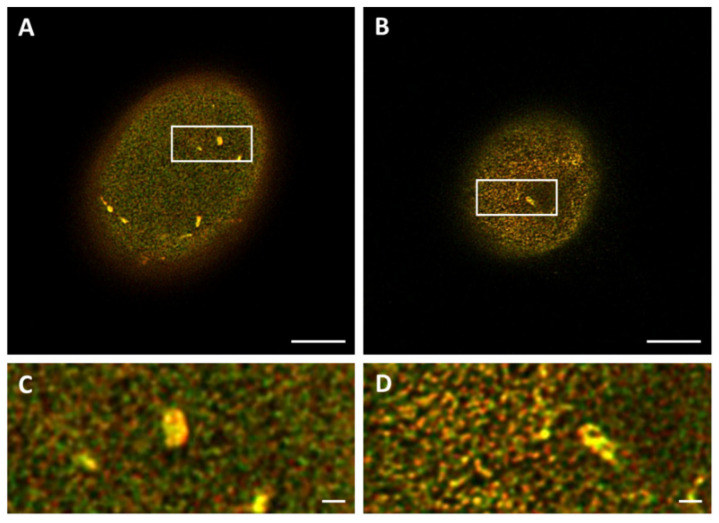
Merged STED microscopy images of the top of the nucleus, visualizing the lamin A (red) and lamin B1 (green) network. (**A**) 3T3 transfected with lamin-A-YFP/lamin-B1-EGFP. (**B**) 3T3 stained with antibodies against lamin A and B1. (**C**,**D**) Higher magnification (4.3x) of ROIs in (**A**,**B**). Scale bars: 5 µm.

The colocalization analysis is performed for STED images taken on top of the nucleus (Figure 6) since this view shows more of the lamin network than images taken at the mid-level of the nucleus. For the transfected 3T3 cells, an average Pearson’s correlation coefficient of 0.74 ± 0.07 (n = 8) was found, while for the 3T3 cells stained with antibodies an average of 0.76 ± 0.02 (n = 7) was found for this score. The difference between the two labeling methods is not significant (*p* = 0.47).

**Figure 7:**
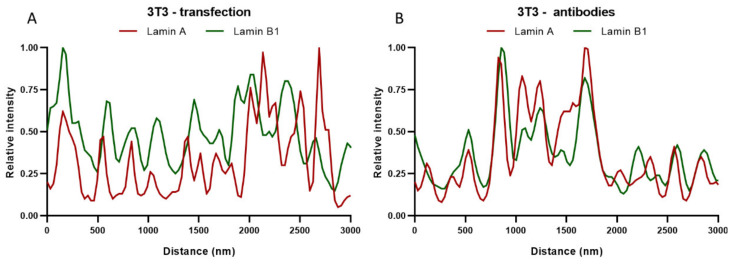
Plot profiles of lamins’ intensities in STED microscopy images, normalized to maximum value. Red curves show the relative intensity of lamin A, green curves the relative intensity of lamin B1. (**A**) 3T3 transfected with lamin-A-YFP and lamin-B1-EGFP. (**B**) 3T3 stained with antibodies against lamin A and B1.

In addition to Pearson’s correlation coefficient, plots of the fluorescence intensities of lamin A and B1 were made by drawing straight lines (ROIs) through the STED images of the top of the nucleus. These intensity plots of lamin A and B1 correspond to their localization in the nuclear lamina. The intensity plots of transfected (Figure 7A) and antibody-stained 3T3 cells (Figure 7B) show a similar profile for both lamin A and B1, with zones of colocalization between lamin A and B1, but also zones with an absence of lamin A or B1. These findings correlate well with the values found for the Pearson’s correlation coefficient, since both do not show a complete colocalization. It is also in line with what can be seen in the STED microscopy images of the top of the nucleus, where also overlapping (yellow) and separate lamin A (red) or lamin B1 (green) spots are visible (Figure 6).

These results, together with the lamin layer thickness analysis, indicate that there are no major differences between transfected and antibody-stained cells. Consequently, the following parts of this microscopy study were performed with the immunostaining approach.

### 2.5. CSLM and STED Colocalization Values

**Figure 8:**
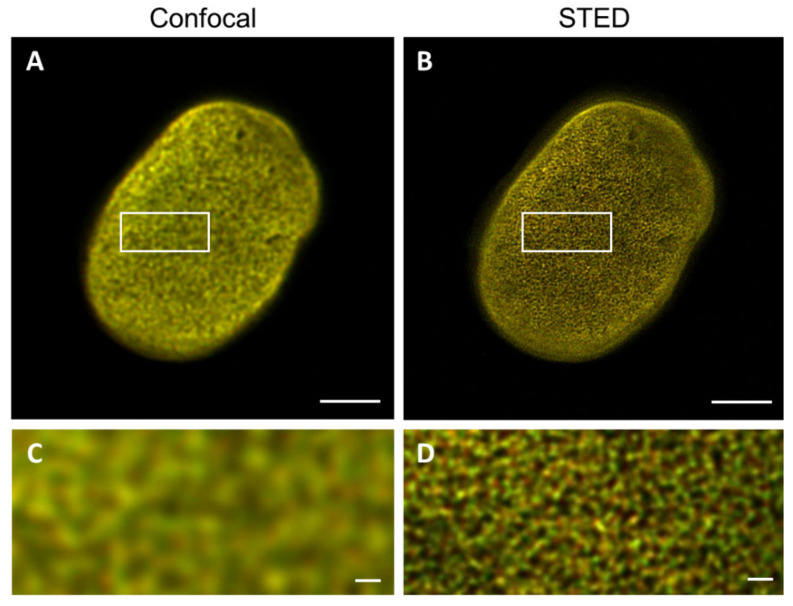
Merged images of the top of the nucleus, visualizing the lamin A/C (red) and lamin B1 (green) network in nHDF cells. (**A**) Confocal microscopy image of nHDF stained with antibodies against lamin A/C and B1. (**B**) STED microscopy image of nHDF stained with antibodies against lamin A/C and B1. (**C**,**D**) Higher magnification (4.3×) of ROIs in (**A**,**B**). Scale bars: 5 µm.

To determine the impact of the resolution difference between CSLM and STED microscopy on the detected degree of colocalization, both confocal and STED microscopy images of the top of nHDF nuclei stained with antibodies against lamin A/C and B1 were analyzed for the degree of colocalization (Figure 8). The average Pearson’s correlation coefficient found for the confocal images is 0.97 ± 0.01 (n = 7) and for the STED images this is 0.86 ± 0.03 (n = 7), demonstrating a significant difference (*p* ≤ 0.0001) between these two microscopic techniques. This finding is not unexpected because a higher resolution is more likely to visualize colocalization differences, as readily visible in the images in Figure 8. Intensity plots of the nHDF (Figure 11A, see below) show a similar course as the intensity plots for 3T3 cells stained with antibodies (Figure 7B), again also supporting the results of the colocalization analysis. The Pearson’s correlation coefficient found for nHDF is increased as compared to 3T3 cells, which is caused by the use of a different primary antibody directed against either lamin A or lamin A/C (133A2 or Jol2, respectively) and possibly by species differences (mouse vs. human cells).

### 2.6. Differences between CSLM and STED Mircoscopy in Imaging Laminopathy Patient Cells

The results described above demonstrated the suitability of using an immunofluorescence protocol for CSLM and STED microscopy of lamin A(/C) and B1 in healthy cells, in which STED microscopy is able to visualize more differences between the two different lamins. To demonstrate the usefulness of the enhanced resolution of STED microscopy in laminopathy patient cells, the lamin layer thickness differences between CSLM and STED microscopy images were also determined for fibroblasts with an *LMNA* c.1130G>T (p.(Arg377Leu)) variant stained with antibodies against lamin A/C and/or lamin B1 (Table 2).

**Figure 9:**
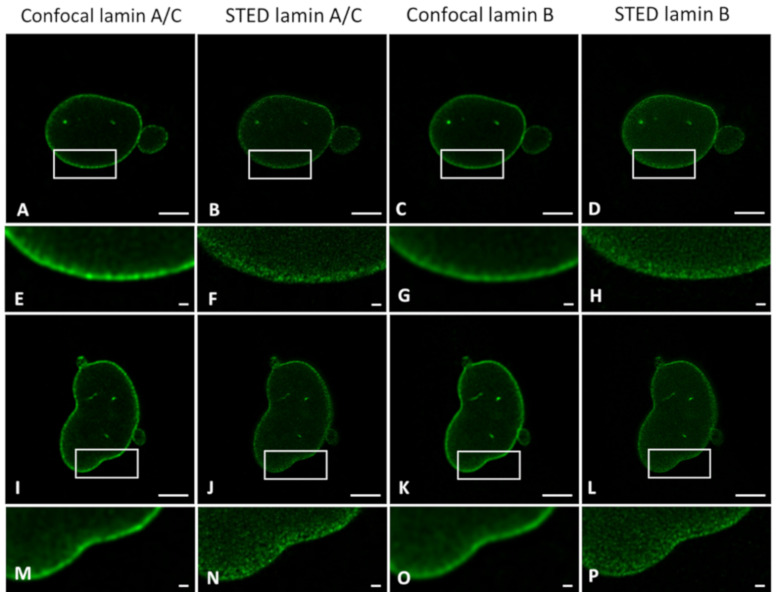
Comparison of confocal (**A**,**C**,**I**,**K**) and STED (**B**,**D**,**J**,**L**) microscopy images of laminopathy patient dermal fibroblasts with an *LMNA* c.1130G>T (p.(Arg377Leu)) variant, stained with antibodies against lamin A/C (**A**,**B**,**I**,**J**) and lamin B1 (**C**,**D**,**K**,**L**). (**E**–**H**) Higher magnification (3.1×) of ROIs in (**A**–**D**). (**M**–**P**) Higher magnification (3.1×) of ROIs in (**I**–**L**). Scale bars: 5 µm.

Importantly, we observed that the lamin layer is not always present as a “sharp line”, but rather as a mesh network in the STED microscopy images, while this is not apparent in confocal images (Figure 9). Due to this, it was not always possible to determine the lamin layer thickness at five positions in each cell, as carried out with the 3T3 cells and nHDF (i.e., the mesh network results in multiple small peaks in the intensity plot, with no possibility of accurate FHWM determination), so sometimes less positions per cell have been measured.

Similar to what was found for the 3T3 cells and nHDFs, the lamin layer thickness found in these laminopathy cells with STED microscopy images is much smaller compared to those found in confocal images, in agreement with the significantly improved resolution of STED. The lamin layer thickness in the laminopathy patient dermal fibroblasts reveals a few small, but statistically significant, differences when compared to the thickness of the nHDF lamina. In the separate staining, the lamin A thickness is found to be significantly (*p* ≤ 0.01) larger as compared to nHDF, but only in CSLM images. The lamin B thickness is significantly larger in both CSLM (*p* ≤ 0.05) and STED (*p* ≤ 0.001) images. However, the co-staining reveals no significant differences for lamin A thickness for both CSLM and STED, while the lamin B thickness is found to be significantly larger in CSLM (*p* ≤ 0.01) and STED (*p* ≤ 0.0001) microscopy images, compared to nHDF.

**Figure 10:**
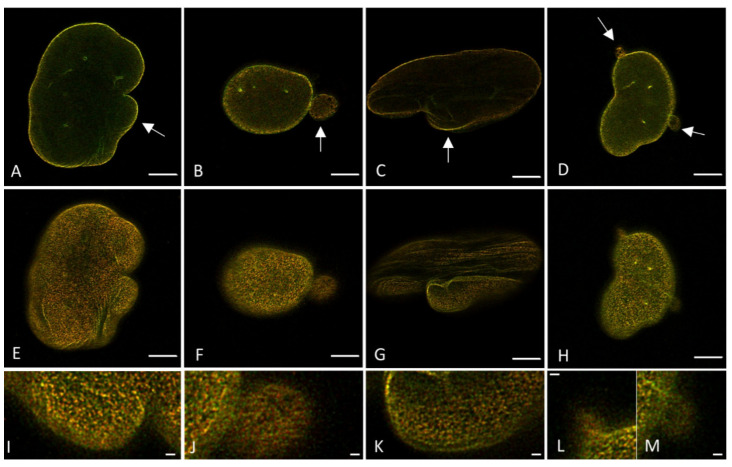
Laminopathy patient dermal fibroblasts with an *LMNA* c.1130G>T (p.(Arg377Leu)) variant, stained with antibodies against lamin A/C and lamin B1, imaged with STED microscopy. (**A**–**D**) Merged image of lamin A/C (red) and lamin B1 (green), mid-level of the nucleus. Arrows: typical laminopathy cell aberrations. (**E**–**H**) Merged image of lamin A/C (red) and lamin B1 (green), top of the nucleus. (**I**–**M**) Higher magnification (3×) of lamin aberrations in (**E**–**H**). Scale bars: 5 µm.

To get an impression of whether or not the degree of colocalization between the A- and B-type lamin network differs in laminopathy patient dermal fibroblasts and if this can be quantified by both CSLM and STED microscopy, fibroblasts with an *LMNA* c.1130G>T (p.(Arg377Leu)) variant stained with antibodies against lamin A/C and/or lamin B1 were imaged with confocal and STED microscopy (Figure 10 and Figure S4). These images already show some readily visible lamin aberrations typical for laminopathy cells, as indicated by the white arrows.

The Pearson’s correlation coefficient determined in confocal images is 0.91 ± 0.03 (n = 6). Compared to the Pearson’s correlation coefficient found in confocal images of nHDF (0.97 ± 0.01), this represents a significant difference (*p* = 0.003), which is already detectable with confocal microscopy. However, colocalization analysis of STED images (e.g., Figure 10E–H) results in an average Pearson’s correlation coefficient of 0.75 ± 0.04 (n = 7). Compared to the Pearson’s correlation coefficient found in STED images of nHDF (0.86 ± 0.03), this is again significantly lower (*p* = 0.00007). Note that the differences between nHDF and the *LMNA* mutant variant found with STED are much larger than those found with confocal microscopy, stressing the advantage of STED microscopy over confocal microscopy for determining differences in lamin A/C and B1 colocalization. Table 3 describes the differences in the Pearson’s correlation coefficient when using a confocal or STED microscopy image.

**Figure 11:**
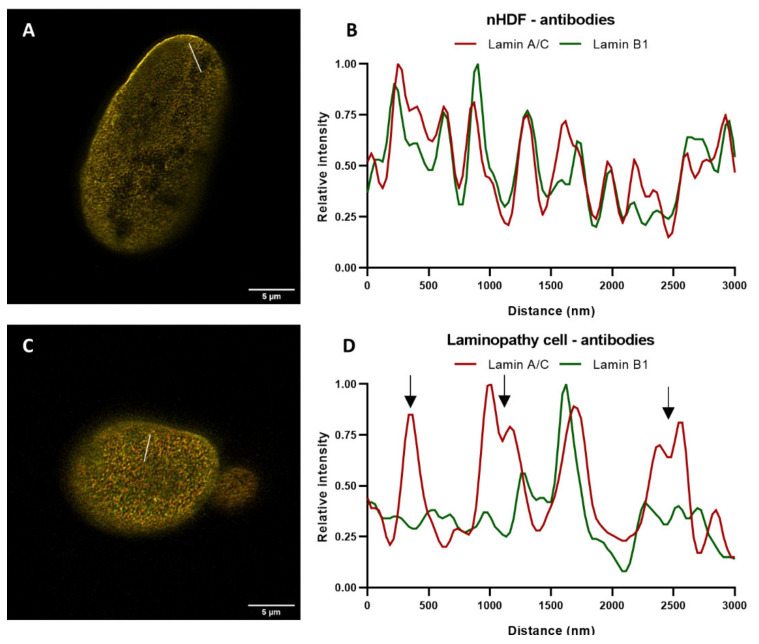
Comparison of nuclear staining patterns between one nHDF (**A**) and a laminopathy patient dermal fibroblast with an *LMNA* c.1130G>T (p.(Arg377Leu)) variant (**C**). Both nuclei were stained with antibodies against lamin A/C and B1. The corresponding intensity plots of ROIs (straight white lines) in (**A**,**C**) are displayed in (**B**,**D**), respectively. Scale bars: 5 µm. (**B**,**D**) Plot profile of lamins’ intensities in STED microscopy images, normalized to maximum value. Red curves show the relative intensity of lamin A/C, green curves the relative intensity of lamin B1. Arrows in (**D**) indicate regions with major segregations between the lamin A/C and lamin B1 network, that are found to a much less extent in the nHDF nucleus (**B**).

In order to further zoom into pattern differences between the lamin A/C and B1 network in apparently normal regions of the nucleus, nuclei of normal and mutant cells, both with the same antibodies, were compared by drawing a straight line (ROI) through the STED image of the top of a nucleus and measuring the intensity (Figure 11A,C).

The intensity plots demonstrate several additional differences between the distribution of lamin A/C and B1 intensity (Figure 11D), compared to nHDF (Figure 11B). The arrows in Figure 11D indicate zones with major differences in lamin A/C and B1 intensity, which corresponds to their localization in the nuclear lamina. These differences were confirmed by the decreased Pearson’s correlation coefficients. These findings indicate that there is enhanced segregation between the A- and B-type lamin networks in this *LMNA* variant. This enhanced segregation could aid in distinguishing normal from diseased nuclei of laminopathy patients.

## 3. Discussion

To better understand the mechanisms of how lamins contribute to disease, it is important to study the lamin network in both healthy and diseased cells. The resolution of ~60 nm of STED microscopy as used to study the nuclear lamina meshwork still lies significantly above its actual thickness. According to cryo-electron tomography studies, the lamina thickness is about 14 nm [7,8]. A recent study using MIET-FLIM confirmed these findings, by proposing a model of the nuclear lamina in which the lamin B1 protein layer has a thickness of ~5 nm and the lamin A/C layer of ~10 nm [46]. Even though the resolution of STED is not as high as the, by other microscopy techniques, estimated lamin thickness, it can still be used to obtain high-resolution information about for instance changes in the lamin network in disease. We compared the differences of using a transfection or immunofluorescence staining approach to study the lamin network with STED microscopy.

Antibody staining was not found to influence, within the resolution limit, the observed lamin layer thickness when compared to transfection. Indirect immunofluorescence labeling attaches a large complex to the structure of interest, but the fluorophore attached to the secondary antibody is the fluorescent signal that is detected with the microscope. Hence, with imaging, not the entire antibody complex is measured, but only the fluorophore attached to the secondary antibody, which can be compared with the size of a fluorescent protein. However, in immunofluorescence labeling multiple antibody complexes are attached to the structure of interest. In the case of lamin labeling, it would, in theory, be possible that one antibody complex is attached to one site of the lamin and another antibody complex to the opposite site. The large distance between antigen and fluorophore on both sides of the lamin would then lead to fluorescent signals quite far apart from each other, visible as a broad lamin layer and a large FWHM. Transfection constructs are incorporated into the original lamina and have a fluorescent protein attached to the lamina, hence having the fluorescent probe located more closely to the lamina. However, imaging the cross-section of fluorescently labeled lamins (when imaging at the mid-level of the nucleus, e.g., Figure 3 and Figure 4) demonstrated no difference in the lamin layer thickness between co-stained and co-transfected cells, therefore suggesting that either method is suitable for visualizing the lamin layer at this resolution level.

Comparison of colocalization analysis of confocal and STED images showed a much higher average Pearson’s correlation coefficient for the confocal images, again demonstrating the potential of STED to distinguish lamin A and B1 networks separately in a co-stained cell. For the 3T3 cells, no significant difference was found between the Pearson’s correlation coefficient of the transfected cells and cells stained with antibodies. However, the standard deviation is higher for the transfected cells. With transfection, the lamin construct is built into the original lamina, which could probably lead to less homogenic signal, because the lamin construct can only be incorporated at places that are accessible (i.e., if there is less original lamina at a certain place more lamin transfection construct can be deposited and vice versa). This could lead to a larger variety in the degree of colocalization. Indeed, the plot profile of transfected 3T3 cells shows fewer overlapping peaks compared to the plot profile of antibody-stained 3T3 cells (Figure 7A,B).

The found values for the Pearson’s correlation coefficient together with lamins’ intensities plot profiles indicate that there is a quite large, but not complete, colocalization of the A- and B-type lamin network within the current resolution limit. Other recent super-resolution studies also investigated the correlation between lamin A and B networks [43,44,45]. Most of these studies did not express the degree of colocalization, but the study of Nmezi et al. did [44]. They reported, by using dSTORM, that the lamin A and B networks are mostly separated at the nuclear surface, with a colocalization of 18%. The reported percentage for colocalization does not correspond with the degree of colocalization found in this study, but this was also determined with another program (Clus-Doc instead of Huygens Professional) and is not expressed with the Pearson’s correlation coefficient, a different microscopy technique was used, and different cells were studied (MEFs). Therefore, these values cannot be accurately compared. Zhironkina et al. [58] showed plot profiles of lamins’ intensities in SIM images taken at the mid-level of the nucleus. These profiles visualize a similar lamin A and B1 distribution, with zones of good colocalization along with gaps and areas, similar as found in this current study. However, immunoelectron microscopy indicated that there are likely no gaps in the lamina meshwork, but rather zones with much lower concentrations of lamins. The authors explain the differences in lamin distribution patterns visualized with different methods by the higher sensitivity of immunoelectron microscopy and an enhanced axial resolution.

As described, the resolution that we used to study the nuclear lamina thickness is above its actual thickness that was estimated between 5 and 10 nm [46]. The diameter of lamin tetramers, which can be made up of distinct types of lamin molecules, is even smaller (~3.5 nm) [7,8]. Nevertheless, studies using PALM and dSTORM, with a resolution (~20 nm) slightly above the thickness of the lamina meshwork, or 3D-SIM, with an even worse resolution (~100 nm), demonstrated distinct networks of lamin A and B. This suggests the formation of lamin homotetramers and not heterotetramers. The colocalization found in the current study, therefore, also does not imply the presence of heterotetramers, but rather the localization of lamin A and B1 close to each other, possibly because of interactions between the two lamin types.

Noteworthy, these above-mentioned super-resolution studies also applied immunofluorescence staining, but do not show a comparison with other labeling strategies and the impact of this on the resolution. One of these studies even demonstrated a localization difference of lamin A/C and lamin B1 in the nuclear envelope, with lamin B1 localized closer to the INM and lamin A/C closer to the nucleoplasm [44]. These results were confirmed by MIET-FLIM, a super-resolution technique that allows a 2.5 nm resolution within a distance range of more than 100 nm, by tracking the fluorescence lifetime of dyes in the near field of a metal film. The shorter the distance to the metal surface, the stronger the reduction in fluorescence lifetime [46]. This provides additional evidence for the ability of antibodies to visualize small localization differences and thereby its potential for super-resolution techniques.

Taken together, we showed that the use of antibodies does not affect the lamina thickness and degree of colocalization of A- and B-type lamins within the resolution of our STED experiments. Using antibodies instead of transfection is especially very convenient when studying laminopathy patient dermal fibroblasts.

Analyzing the colocalization in laminopathy patient fibroblasts, stained with antibodies against lamin A/C and B1, revealed a significantly decreased colocalization of lamin A/C and B1 in both confocal and STED images, compared to nHDF. However, this decrease is more obvious when utilizing STED microscopy. These findings confirm the ability of STED microscopy to visualize more differences between lamin A/C and B1 in comparison to CSLM. The described findings suggest enhanced segregation between the A- and B-type lamin networks in laminopathy. Future studies should reveal if the decreased lamin A/C and B1 colocalization is common in different laminopathy patient fibroblasts. When future studies in laminopathy patient fibroblasts indeed reveal a common decrease in lamin A/C and B1 colocalization in multiple *LMNA* variants, this could contribute to the biopsy-based diagnosis of laminopathy. Nowadays classification of laminopathy is performed based on a significant number of abnormal nuclei in fibroblast. However, patients with an *LMNA* mutation only show 10–25% dysmorphic nuclei, while a normal nuclear morphology does not rule out pathogenicity of the *LMNA* variant [37,38].

The lamin B1 layer thickness in the laminopathy patient fibroblasts appeared to be significantly larger in both confocal and STED images of separate and co-stained cells, while for lamin A/C only in confocal images of separately stained cells a significant difference was found. To determine if the thicker lamin B1 layer might be a sort of compensation mechanism, future studies should also examine the lamin layer thickness of a multitude of laminopathy patient fibroblasts.

In conclusion, this study revealed that super-resolution light microscopy in combination with immunofluorescence protocols is an excellent tool for this novel research approach to study laminopathy patient fibroblasts. In addition, the resolution that can be accomplished with super-resolution light microscopy will continuously increase in the coming years, leading to exciting new opportunities to obtain more information about, amongst others, the nuclear lamina network.

## 4. Materials and Methods

### 4.1. Cell Culture

3T3 mouse fibroblasts, normal human dermal fibroblasts (nHDF), and laminopathy patient dermal fibroblasts (see below) were cultured in Dulbecco’s Modified Eagle Medium (DMEM) (Gibco, Thermo Fisher Scientific Inc., Waltham, MA, USA) containing 10% fetal calf serum (FCS) (Gibco) and 50 µg/mL Gentamycin (Dechra, Northwich, UK). The cells were incubated at 37 °C and 5% CO_2_ in a humidified incubator. At confluence, the cells were trypsinized using 0.05% Trypsin/0.02% EDTA/0.02% glucose solution in phosphate-buffered saline (PBS) and passaged by splitting in a 1:5 or 1:10 ratio for 3T3 cells, 1:2 or 2:3 ratio for dermal fibroblasts. The dermal fibroblasts were fixed and stained at passage number p11–p15.

The laminopathy patient dermal fibroblasts (*LMNA* c.1130G>T (p.(Arg377Leu))) were obtained from a skin biopsy of a 40-year-old donor, who previously underwent heart transplantation after several cardiac arrests resulting from heart failure due to Dilated Cardiomyopathy (DCM). During his lifetime he had developed increasing arrhythmia complaints. Several family members died of heart failure due to DCM. The DCM status was confirmed by histological analysis of the removed heart. Screening of the fibroblast culture by regular immunofluorescence microscopy revealed nuclear abnormalities, including characteristic honeycomb structures, and herniations, in more than 10% of the cells, confirming the pathologic status of these cells (for criteria see [37], (Veltrop et al. in preparation).

For live cell imaging with STED, cells were cultured in a µ-Slide 2 Well (IbiTreat #1.5, Ibidi GmbH, Gräfeling, Germany) in DMEM without phenol red (Gibco) containing 10% FCS and 50 µg/mL Gentamycin.

### 4.2. Transfection and Subcloning

3T3 wildtype cells were transfected with lamin-A-YFP (Yellow Fluorescent Protein) and/or lamin-B1-EGFP (Enhanced Green Fluorescent Protein) using FuGENE HD Transfection Reagent according to the manufacturer’s instructions. Lamin-A-YFP was obtained by cloning the lamin A fragment from lamin-A-EGFP [14] into the YFP-C1 vector (Clontech Laboratories Inc., Palo Alto, CA, USA). After 4 h, the culture medium was discarded, and new medium was added. One day later, selection for stable transfectants was started by adding Geneticin (50 mg/mL, Gibco) to the culture medium (500 µg/mL). Approximately 10 days later, cells were subcloned to single-cell colonies by limited dilution. GFP/YFP-expressing colonies were selected using a widefield fluorescence microscope (ZEISS Axiovert 35M). At sufficient cell growth, selected colonies were transferred to a 6-wells plate and thereafter a T25 flask.

### 4.3. Cell Fixation and Immunofluorescence Staining

Cells were seeded onto glass coverslips (#1.5), grown for two days, and fixed with formaldehyde (4% in PBS) at room temperature (RT) for 15 min or with methanol at −20 °C for 10 min. Formaldehyde fixed cells were permeabilized in Triton X-100 (0.1% in PBS) for 15 min at RT prior to antibody staining. Primary antibodies were diluted in PBS containing 3% bovine serum albumin (BSA, Roche Diagnostics, Basel, Switzerland), applied onto the coverslips, and incubated for 1 h at RT. The primary antibodies used were:

(1) mouse monoclonal IgG1 anti-lamin A/C culture supernatant (Jol2; 1:50; kindly provided by Prof. C. Hutchison, Durham, UK),

(2) mouse monoclonal IgG3 anti-lamin A culture supernatant (133A2; 1:50; Nordic-MUbio, Susteren, The Netherlands), and

(3) rabbit polyclonal IgG anti-lamin B1 (ab16048; 1:1000; 1 mg/mL; Abcam plc, Cambridge, UK).

After washing in PBS, secondary antibodies (diluted in PBS containing 3% BSA) were applied for 1 h at RT. The secondary antibodies used were:

(1) Goat anti-mouse IgG Abberior STAR GREEN (1:1000; 1 mg/mL; Abberior Instruments GmbH, Göttingen, Germany), and

(2) Goat anti-rabbit IgG Abberior STAR 512 (1:500; 1 mg/mL; Abberior Instruments GmbH, Göttingen, Germany).

A final washing step in PBS was performed before the coverslips were mounted on an object glass with Tris-Glycerol DABCO mounting medium (90% glycerol, 20 mM Tris-HCl pH 8.0, 2% 1,4-di-azobicyclo-2(2,2,2)-octane (Merck, Darmstadt, Germany), and sealed with nail polish.

The 3T3 cells transfected with lamin-A-YFP and/or lamin-B1-EGFP were directly mounted on a cover glass after fixation with formaldehyde or methanol, as described above.

### 4.4. CSLM and STED Microscopy

Both confocal and STED images of the fixed cell and live samples were obtained with the Leica TSC SP8 STED microscope using LAS X software (version 3.5.5.19976, Wetzlar, Germany). Live cell imaging was performed by incubation at 37 °C and 5% CO_2_. All imaging was carried out with the HC PL APO 86x/1.20 W motCORR objective and the HyD detector. The refractive index was adjusted with the correction collar; to that end, the collar position was adjusted to the value that gives the brightest fluorescence signal. Imaging was carried out in acquisition mode xyz, with a gating of 0.2–7.0 ns, format of 1024 × 1024 pixels, speed of 400 Hz, and pixel size of around 30 × 30 nm. Fixed cells were imaged in photon counting mode, with a gain of 10%, frame average of 1, and line accumulation of 8 for confocal images and 16 for STED images. Live cells were imaged in standard mode, with a gain of 100%, frame average of 3, and line accumulation of 1 for confocal images and 2 for STED images. For all samples, 50% STED power was used. Cells transfected with YFP were imaged with white light laser excitation at 513 nm and detection of emission at 523–560 nm, cells transfected with EGFP with 483 nm and 489–525 nm respectively. Cells stained with the secondary antibody Abberior STAR GREEN were imaged with excitation at 488 nm and emission detection at 493–545 nm, Abberior STAR 512 at 521 nm and 526–565 nm, respectively. In the case of two fluorophores per sample, sequential imaging was applied. For all images, a small Z-stack of three slides (step size 0.10 µm) was generated.

### 4.5. Image Analysis

After image acquisition, all images were deconvoluted with Huygens Professional version 19.10 (Scientific Volume Imaging, Hilversum, The Netherlands, https://svi.nl/HowtoCiteHuygens (accessed on 13 September 2021)), using the classic maximum likelihood estimation (CMLE) algorithm, with 40 maximum iterations and a calculated signal-to-noise ratio (SNR) (SNR = √((maximum value histogram image)/(lowest value histogram image)) ∗ 3). The other options were left at default settings.

The FWHM was measured using Fiji [59]. To estimate the lamin layer thickness, the average FWHM of the lamina was determined at the mid-level of the nucleus. For the quantitative analysis, the layer thickness was determined at five positions in each cell. Intensity curves were plotted through lines drawn perpendicular to the lamina and fitting this with a Gaussian distribution. The formula 2√2(ln2σ), with σ as Gaussian width parameter, was used to calculate the FWHM [60]. To validate that this method of layer thickness determination did not overestimate the effective layer thickness, which is described by Tortarolo et al. as a possible disadvantage of this method, the FWHM determination was performed by two different persons in an independent manner [46]. The second determination gave comparable results, with the average FWHM value of the second determination within the standard deviation of the average FWHM value of the first determination.

To determine the degree of colocalization of cells transfected with lamin-A-YFP and lamin-B1-EGFP or stained for lamin A(/C) and B1, Pearson’s coefficient value was determined using the Colocalization Wizard in Huygens Professional (threshold settings: Costes method). For statistical analysis of the found values, the Student’s t-test was performed (two-sided, unequal variances). *p*-values ≤ 0.05 were considered statistically significant (* *p* ≤ 0.05, ** *p* ≤ 0.01, *** *p* ≤ 0.001, **** *p* ≤ 0.0001).

## Figures and Tables

**Table 1:** Average STED and confocal microscopy FWHM ± SD (nm) as a measure of the lamin layer thickness of lamin A(/C) and B1 in transfected 3T3 cells, 3T3 cells stained with antibodies, and nHDF stained with antibodies, both in separate transfection or immunostaining and co-transfection or co-staining. The layer thickness was determined at five different positions in each cell.

Cell Type, Labelling Condition	Lamin A(/C) STED	Lamin A(/C) Confocal	Lamin B1 STED	Lamin B1 Confocal
3T3, transfection	132 ± 15 (n = 7)	286 ± 20 (n = 7)	144 ± 37 (n = 7)	297 ± 18 (n = 7)
3T3, co-transfection	140 ± 26 (n = 10)	322 ± 22 (n = 10)	151 ± 26 (n = 10)	309 ± 22 (n = 10)
3T3, antibody staining	145 ± 20 (n = 7)	291 ± 19 (n = 7)	146 ± 21 (n = 9)	311 ± 13 (n = 9)
3T3, antibody co-staining	120 ± 27 (n = 7)	275 ± 16 (n = 7)	123 ± 19 (n = 7)	303 ± 24 (n = 7)
nHDF, antibody staining	132 ± 24 (n = 7)	290 ± 25 (n = 7)	138 ± 18 (n = 7)	307 ± 32 (n = 7)
nHDF, antibody co-staining	125 ± 28 (n = 7)	293 ± 20 (n = 7)	112 ± 22 (n = 7)	291 ± 23 (n = 7)

**Table 2:** Average STED and confocal microscopy FWHM ± SD (nm) as a measure of the lamin layer thickness of lamin A/C and B1 in laminopathy patient dermal fibroblasts with an *LMNA* c.1130G>T (p.(Arg377Leu)) variant, stained with antibodies against lamin A/C and/or lamin B1. The layer thickness was determined at 1–5 positions in each cell.

Staining	Lamin A/C STED	Lamin A/C Confocal	Lamin B1 STED	Lamin B1 Confocal
Separate staining	139 ± 29 (n = 7)	311 ± 31 (n = 7)	156 ± 16 (n = 4)	328 ± 28 (n = 4)
Co-staining	136 ± 23(n = 7)	306 ± 36 (n = 7)	150 ± 29 (n = 7)	315 ± 30 (n = 7)

**Table 3:** Comparison of the Pearson’s correlation coefficient, as a measure for lamin A/C and B1 colocalization, found in CSLM and STED images of the same laminopathy dermal patient fibroblasts with an *LMNA* c.1130G>T (p.(Arg377Leu)) variant.

Pearson’s Correlation Coefficient CSLM	Pearson’s Correlation Coefficient STED
0.925	0.758
0.887	0.707
0.873	0.724
0.900	0.761
0.951	0.816
0.908	0.763

## Data Availability

Not applicable.

## Supplementary Materials

The following are available online at www.mdpi.com/article/10.3390/ijms221910194/s1.
