# Supplementary material for: Super-Resolution Imaging of the A- and B-Type Lamin Networks: A Comparative Study of Different Fluorescence Labeling Procedures
Source: Int J Mol Sci. 2021 Sep 22;22(19):10194. doi: 10.3390/ijms221910194 (PMC8508656; doi:10.3390/ijms221910194)
Supplement: Supplementary file 1 [file ijms-22-10194-s001.zip › ijms-1289716-supplementary.pdf]

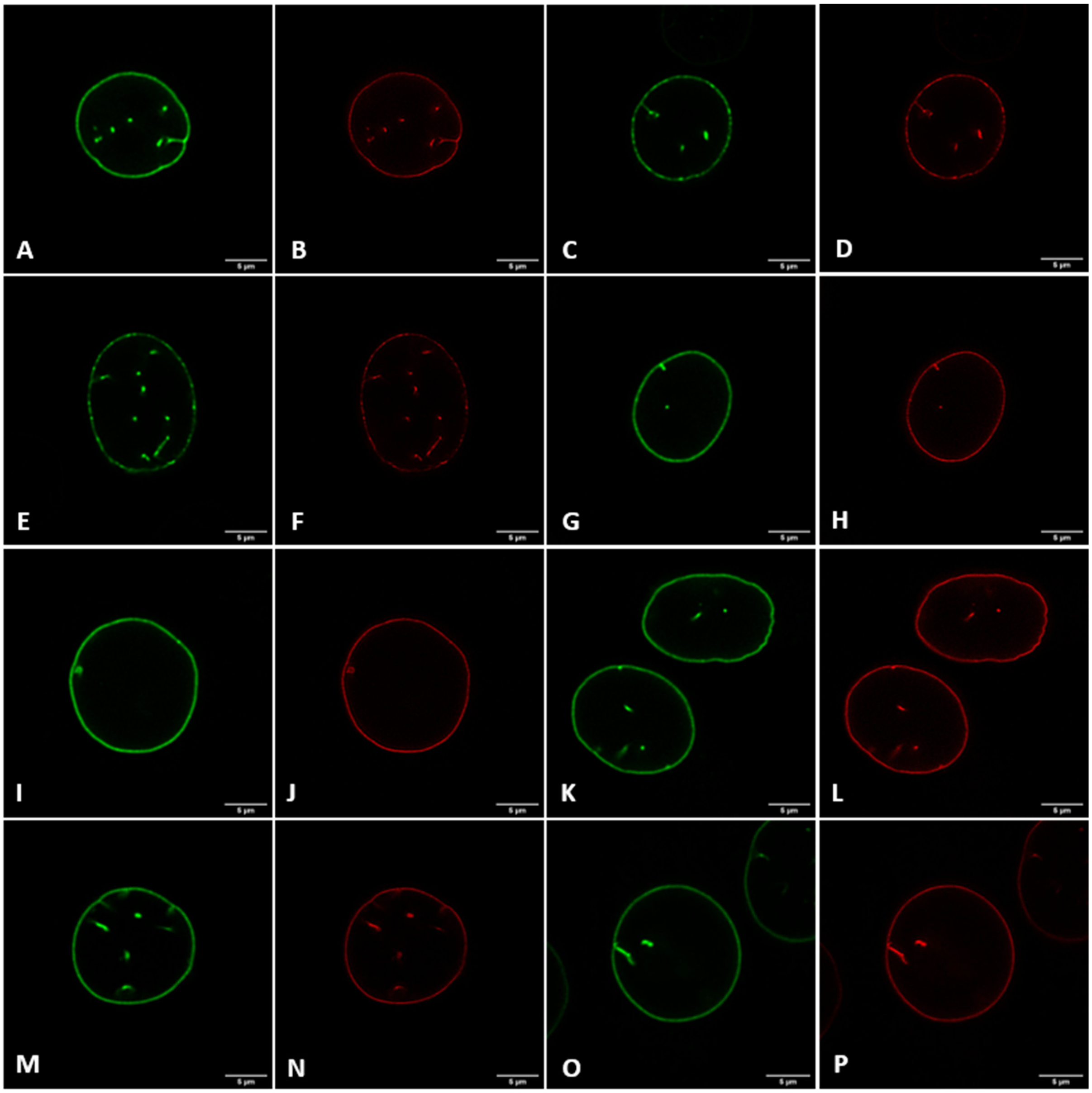

Figure S1: Confocal (green) and STED (red) images of 3T3 cells lamin-A-YFP (**A-H**) or lamin-B1-EGFP (**I-P**) transfected. Scale bars: 5 μM.

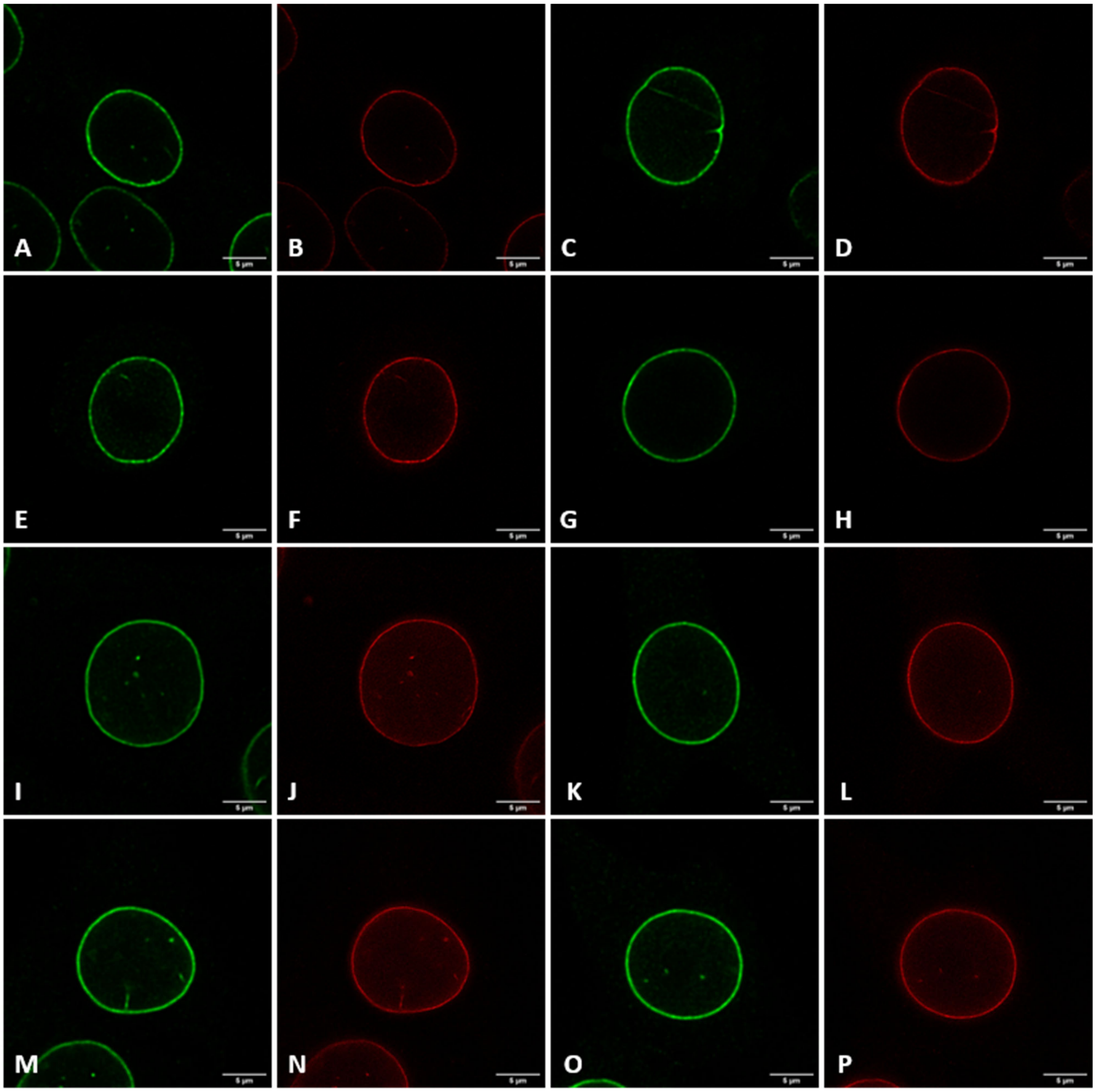

Figure S2: Confocal (green) and STED (red) images of 3T3 cells stained with antibodies against lamin A (**A-H**) or lamin B1 (**I-P**). Scale bars: 5 μM.

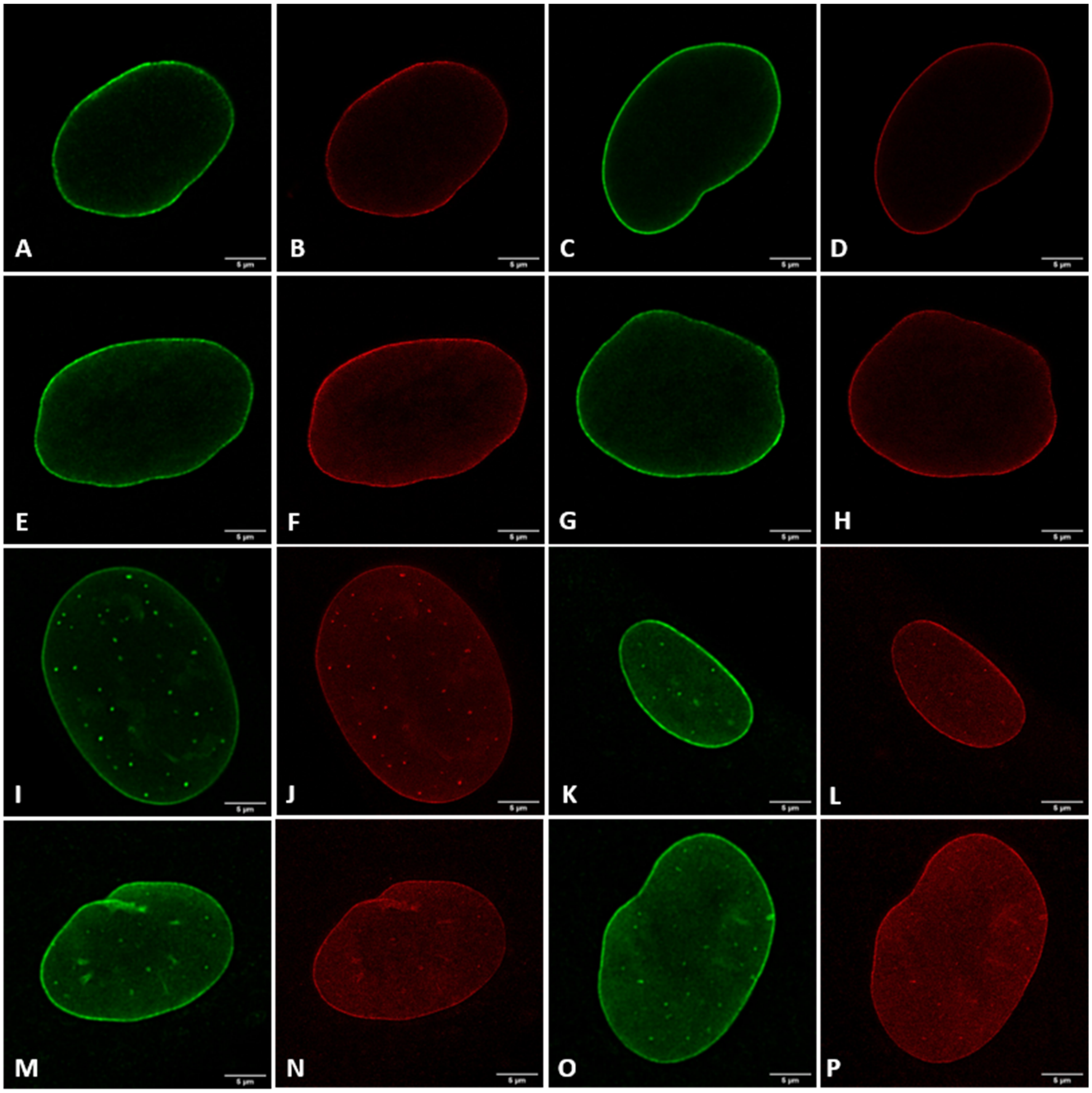

Figure S3: Confocal (green) and STED (red) images of nHDF stained with antibodies against lamin A (**A-H**) or lamin B1 (**I-P**). Scale bars: 5 µM.

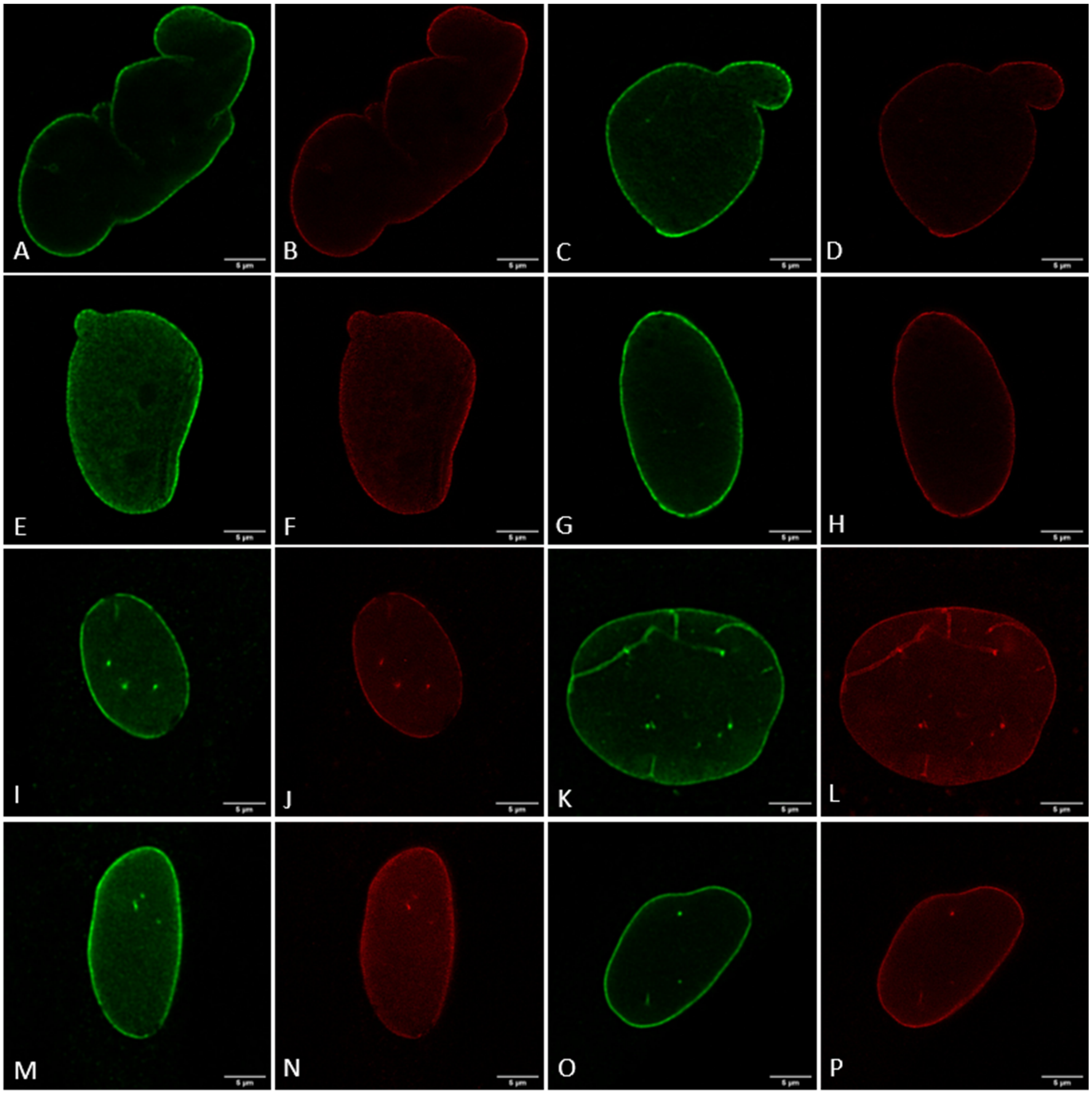

Figure S4: Confocal (green) and STED (red) images of laminopathy patient dermal fibroblasts with a *LMNA* c.1130G>T (p.(Arg377Leu)) variant, stained with antibodies against lamin A (**A-H**) or lamin B1 (**I-P**). Scale bars: 5 μM.
